# Current Status and Future Therapeutic Options for Fecal Microbiota Transplantation

**DOI:** 10.3390/medicina58010084

**Published:** 2022-01-06

**Authors:** Sergii Tkach, Andrii Dorofeyev, Iurii Kuzenko, Nadiya Boyko, Tetyana Falalyeyeva, Luigi Boccuto, Emidio Scarpellini, Nazarii Kobyliak, Ludovico Abenavoli

**Affiliations:** 1 Ukrainian Research and Practical Centre of Endocrine Surgery, Transplantation of Endocrine Organs and Tissues of the Ministry of Health of Ukraine, 01021 Kyiv, Ukraine; tkachsergio@ukr.net (S.T.); yuriykuzenko@ukr.net (I.K.); 2 Shupyk National Medical Academy of Postgraduate Education, 04112 Kyiv, Ukraine; dorofeyevand@ukr.net; 3 RDE Center of Molecular Microbiology and Mucosal Immunology, Uzhhorod National University, 88000 Uzhhorod, Ukraine; ladiya.boyko@uzhnu.edu.ua; 4 Educational-Scientific Center, Institute of Biology and Medicine, Taras Shevchenko National University of Kyiv, 01601 Kyiv, Ukraine; tetyana.falalyeyeva@knu.ua; 5 Department of Histopathology, Medical Laboratory CSD, 03022 Kyiv, Ukraine; n.kobyliak@csd.com.ua; 6 School of Nursing, Healthcare Genetics Program, Clemson University, Clemson, SC 29646, USA; lboccut@clemson.edu; 7 Translational Research Center for Gastrointestinal Disorders (TARGID), Department of Chronic Diseases, Metabolism and Ageing (ChroMetA), Catholic University of Leuven, 3000 Leuven, Belgium; emidio.scarpellini@med.kuleuven.be; 8 Department of Endocrinology, Bogomolets National Medical University, 01601 Kyiv, Ukraine; 9 Department of Health Sciences, University “Magna Graecia”, Viale Europa-Germaneto, 88100 Catanzaro, Italy

**Keywords:** gastrointestinal diseases, liver diseases, health, metabolism

## Abstract

The intestinal microbiota plays an important role in maintaining human health, and its alteration is now associated with the development of various gastrointestinal (ulcerative colitis, irritable bowel syndrome, constipation, etc.) and extraintestinal diseases, such as cancer, metabolic syndrome, neuropsychiatric diseases. In this context, it is not surprising that gut microbiota modification methods may constitute a therapy whose potential has not yet been fully investigated. In this regard, the most interesting method is thought to be fecal microbiota transplantation, which consists of the simultaneous replacement of the intestinal microbiota of a sick recipient with fecal material from a healthy donor. This review summarizes the most interesting findings on the application of fecal microbiota transplantation in gastrointestinal and extraintestinal pathologies.

## 1. Introduction

**Figure 1:**
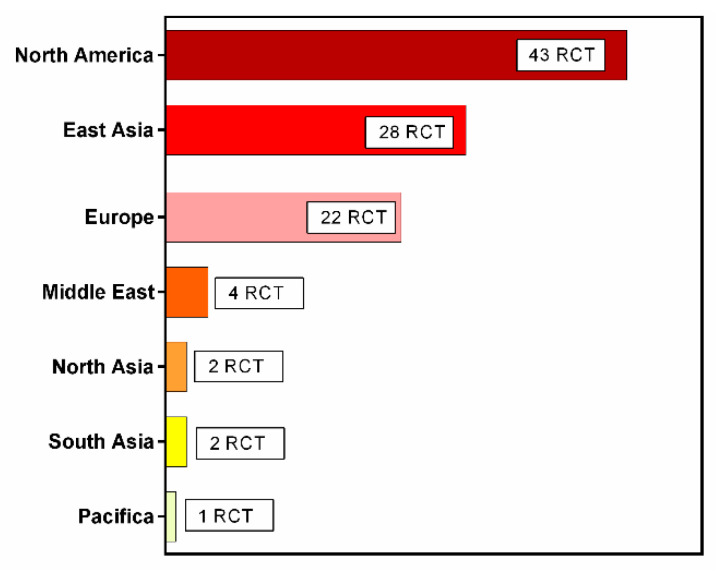
Worldwide distribution of FMT research across regions.

**Figure 2:**
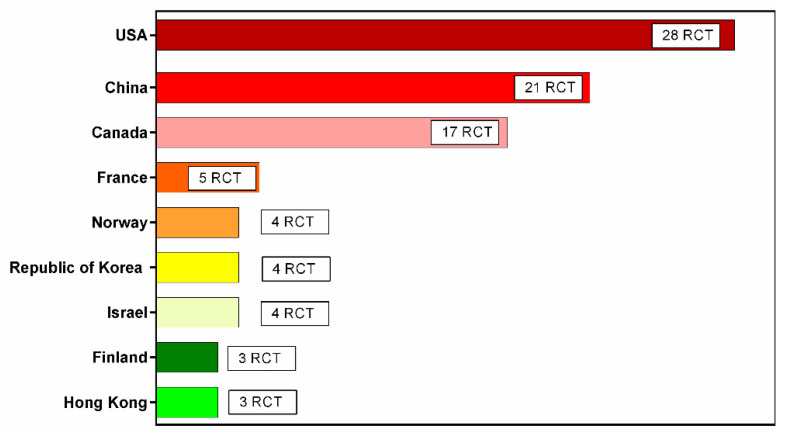
List of countries with the most active contributions in FMT investigation.

The gut microbiota has a critical function in human health, and its various disorders are associated with the development of particular diseases [1,2]. Disruption of the gut microbiota may lead to both gastrointestinal and non-gastrointestinal diseases, such as cancer, metabolic syndrome, or neuropsychiatric diseases [3,4,5,6,7,8,9]. Therefore, it is not surprising that various methods aiming to modify the composition of gut microbiota have always attracted the close attention of both representatives and practitioners in the scientific community. One such method is fecal microbiota transplantation (FMT), which consists of the simultaneous replacement of the gut microbiota of an ill recipient with healthy donor fecal material. Interest in FMT is far from recent development; it was first described by Ge Hong in the treatment of severe diarrhea as early as the 4th century AD [10]. Active participation in the field of contribution for FMT research varies between different countries and geographical regions (Figure 1 and Figure 2). The modern stage of studies on FMT began in 1958 when, for the first time in the scientific literature, American surgeon Ben Eisman described four patients with antibiotic-associated diarrhea, as well as a rapid clinical improvement after the use of enemas with donor fecal material [11]. Nevertheless, it was not officially recognized for over 50 years until the first controlled study was conducted in Amsterdam, which demonstrated the high efficiency of FMT in instances of recurrent infection of *Clostridium difficile* [12]. At present, this pathology is the only officially approved ground for FMT, and is routinely used worldwide [13]. However, the effectiveness of FMT in the therapy of other gastrointestinal and non-gastrointestinal diseases that are not associated with the recurrent infection of *Clostridium difficile* is currently being studied [14].

## 2. Ulcerative Colitis

Most of the studies supporting the effectiveness of FMT for diseases that are not associated with recurrent infections of *Clostridium difficile* relate to inflammatory bowel diseases, such as primarily ulcerative colitis (UC), due to the significant violations of the quantitative and qualitative composition of gut microbiota inherent to this pathology. In particular, the variety of gut microbiota is lower in patients with UC compared with healthy people, and there is an observable decline in the relative amount of *Firmicutes* (*Clostridium* clusters XIVa and IV) and *Bacteroidetes*. It was found that the development of UC is associated with a significant decrease in butyrate produced by *Fecalibacterium prausnitzii*, as well as a significant increase in the numbers of *Proteobacteria* and *Actinobacteria* [15]. As a result of intestinal dysbiosis, there is a decrease in the production of short-chain fatty acids (SCFAs), primarily butyrate, which is considered an essential source of nutrition for colonocytes and plays important role in immune regulation. [16]. There are currently five known randomized controlled trials (RCT) on the effectiveness of FMT in patients with active UC, and three of them demonstrated positive results. Moayyedi et al. studied 70 patients with active UC who participated in single-blind RCT and received either allogeneic FMT by enema (experimental group) or water enemas (control group) [17]. Primary endpoints, such as a decrease in total Mayo score of less than 3 and endoscopic healing (0 on an endoscopic Mayo scale), were recorded in 24% of patients receiving FMT and 5% of patients receiving a placebo for 6 weeks after the start of the RCT. Interestingly, the majority of patients who demonstrated an unusually positive effect received FMT from one super donor (39% as compared to 10% with material from other donors), which confirms the crucial role of donor selection. The authors note that such data are not indicated in the treatment of recurrent *Clostridium difficile* infections. Rossen et al. studied the effectiveness of FMT in 25 patients with active UC (as well as with a control group with other patients who received their own fecal material in the same way, *n* = 25) by carrying out by 2 duodenal infusions consisting of 500 mL of fresh allogeneic fecal material [18]. After 3 weeks, a clinical and endoscopic response was recorded in 30% of patients with UC who had received allogeneic treatment, and 20% of patients who had received autologous fecal material; these differences were insignificant. Paramsothy et al. studied the effectiveness of FMT by administering colonoscopies in patients with mild to moderate UC, and most of the patients received FMT through injections of donor material from 3–7 individuals [19]. Steroid-free remission and an endoscopic response or remission were achieved in 11 of the 41 (27%) patients who received active fecal material and 3 of the 40 (8%) patients who received a placebo (saline). The clinical effectiveness was associated with magnification in gut microbiota variety and the lack of an effect was connected with a relative rise in the representation of *Fusobacterium*. Costello et al. studied the effectiveness of FMT in patients with mild to moderate UC through the repeated administration of frozen fecal material from several donors by enema [20]. The results were comparable to those of the previous study: remission in 32% of patients treated with fecal material, as opposed to remission in 9% of placebo-treated patients. To assess the efficacy and safety of FMT in UC, 25 studies were analyzed in a systematic review. It was established that from more than 200 patients with UC included for analysis, 42% obtained clinical remission (CR) and 65% achieved clinical response. In the cohort studies, the pooled estimate of patients who achieved CR was 41%, and the clinical response was 66%. [21]. Another systematic review with a meta-analysis that included data from 277 participants was performed to estimate FMT as a therapy for active UC [22]. Among four RCT, FMT was connected with higher combined clinical and endoscopic remission, compared with the control (risk ratio UC not in remission was 0.80; 95% CI: 0.71–0.89) with a treatment number of five (95% CI: 4–10) [22]. Thus, in a certain proportion of patients with UC, FMT has demonstrated its clear advantages over a placebo. However, its effectiveness seems to depend on the method of donor material administration and the donor material used (from one or several donors, from a normal healthy donor or a super donor, fresh material or frozen material), as well as the number of procedures (a single FMT or repeated sessions), previous treatment, and UC severity. Until now, it was unknown which microbes or microbial components mediate the treatment action of FMT in patients with UC.

## 3. Pouchitis

Pouchitis is the most widespread sequela in patients who undergo ileal pouch-anal anastomosis, occurring more frequently in patients with UC. Pouchitis—the inflammation of the pouch—can be due to idiopathic or secondary causes [23]. The most difficult forms to treat are chronic antibiotic-dependent pouchitis (CADP) and chronic antibiotic-resistant pouchitis (CARP) [23]. The efficacy of antibiotics in pouchitis makes manipulation of the gut microbiota an attractive option to explore as a potential therapeutic pathway [24]. Promising new treatments for difficult-to-treat forms of chronic pouchitis, such as FMT, show encouraging preliminary results in small studies, according to some reports. The initial study administered a single FMT via a nasogastric tube to rifaximin-free for 2 weeks patients with CARP; of the eight patients enrolled, none achieved clinical remission. Despite this, two patients with previous ciprofloxacin resistance became sensitive to the antibiotic and showed the reduction of PDAI drop ≥ 3 following FMT [25]. Selvig et al. reported the largest pilot prospective open-label study, including 19 patients with chronic pouchitis, undergoing single FMT by pouchoscopy from healthy, screened donors. While post-FMT PDAI scores were not accessible for all cases, and some had PDAI scores ≤ 6 pre-FMT, the endoscopic and histologic results did not decrease significantly post-FMT [26]. Nevertheless, there was a statistically significant betterment in gut motility, and a trend for improvement in abdominal pain post-FMT [26]. Stallman et al. fractionally supported these data, reporting an endoscopic response in all persons and an endoscopic remission in one patient, following FMT infusions [27]. There is currently a paucity of available data and a lack of RCT about FMT for pouchitis. In the only double-blind RCT to date—in which participants were randomized to a single colonoscopic-delivered FMT followed by oral capsules versus placebo—the investigation was stopped prematurely, due to low donor FMT engraftment and the absence of clinical responses [28]. Available data suggest that the application of antibiotics pre-FMT in UC patients assists in the engraftment of beneficial *xenomicrobiota*, improving the response of treatment [29]. The use of antibiotics pre-FMT is promising, since antibiotic therapy is the primary therapy modality in pouchitis. Nevertheless, this is only if an adequate selection of antibiotics, dosage, and period of therapy are ensured [30]. Moreover, much focus was given to changes in the diversity of the pouch microbiome. It was found that successful multiple FMT in patients with UC and pouchitis was accompanied by an increase in *Firmicutes* and a decrease in *Proteobacteria* in the ileal pouch [31]. In the study of Selvig et al., microbiota profiling revealed no distinct community-level changes post-FMT, but a small number of specific bacterial taxa significantly differed in relative abundance [26].

The data on the efficacy of FMT in pouchitis from three small cohort studies were firstly summarized in sub-analysis as a part of a comprehensive meta-analysis in IBD subtypes [32]. Analyzed studies were descriptive, with different FMT infusion regimens, endpoints, and conflicting outcomes. The reported clinical remission rate following FMT was 21.5% [32]. The most recent pouchitis-specific systematic review with meta-analysis included 9 investigations, reporting the FMT treatment of 69 patients. Generally, clinical effectiveness after FMT was reported in 32% of the 44 evaluated patients at various time points after FMT, and clinical remission in 23% of the patients [33].

Future larger RCTs with a well-controlled design to estimate the efficacy and safety of FMT for pouchitis are warranted. There are also open issues that need to be resolved, such as the optimal dosing regimen (interval and frequency of infusions), and if there are any benefits to employing multi-donor/pooled infusions, as well as preselecting donors according to microbial profiles, etc. [30].

## 4. Crohn’s Disease

Considering the positive results of FMT in patients with UC, its effectiveness has also been studied in patients with Crohn’s Disease (CD). However, the evidence for the effectiveness of FMT in patients with CD is significantly less compelling. Several open-label studies have been published with mixed results, but there have been no controlled studies on the effectiveness of FMT in patients with CD [34,35]. Vermeire et al. found that there was no remarkable improvement among the six patients with CD at week 8, following FMT via ileocolonoscopy. However, one person experienced interim clinical remission for 1.5 months. A recent study with an open-label design included 30 patients with refractory CD (Harvey–Bradshaw Index (HBI) score ≥ 7) for single FMT through mid-gut, who were assessed during follow-up. The rate of clinical improvement and remission based on clinical activity at the first month was 87% and 77%, accordingly, which was greater than other evaluation points within a 15-month follow-up. FMT also appeared to have a fast and continuous significant effect in relieving the sustaining abdominal pain associated with sustaining CD [35]. Nine adolescent patients with mild-to-moderate symptoms defined by the Pediatric Crohn’s Disease Activity Index (PCDAI of 10–29), were enrolled into a prospective open-label study of FMT through a nasogastric tube, with check-up assessments at 2, 6, and 12 weeks. The mean PCDAI score improved in patients: with betterment by 68% at 2 weeks and 56% at 6 weeks. Based on PCDAI, 7 of the 9 patients were in remission at 2 weeks, and 5 of the 9 patients who did not receive extra medical treatment were in remission at 6 and 12 weeks. In patients who did not engraft, or whose microbiome was similar to their donor, improvement was not seen [36]. The efficacy of FMT was estimated in a meta-analysis with the inclusion of 39 CD patients. CR and/or mucosal healing were identified as primary outcomes. Analyses have shown a pooled evaluation of CR of 61% for CD [37]. The most recent cumulative meta-analysis summarized data from available 2020 studies for both CD and UC. The comparative analyses exposed that frozen fecal material from universal donors may be connected with a higher rate of CR, especially for CD. Pairwise meta-analyses of six RCT showed significant differences in favor of fecal microbiota transplantation compared with the placebo (clinical remission: RR 1.70 [95% CI 1.12, 2.56]; clinical response: RR 1.68 [95% CI 1.04, 2.72]). The interventional studies found an overall clinical remission of 37%, and an overall clinical response of 54% [38]. Sokol et al. performed a first randomized, single-blind, sham-controlled pilot trial (NCT02097797) of FMT in adults with colonic or ileocolonic CD [39]. The primary endpoint was the implantation of the donor microbiota at week 6 (Sorensen index > 0.6). None of the patients reached the primary endpoint. The steroid-free clinical remission rate at 10 and 24 weeks was 44% and 33% in the sham transplantation group, and 88% and 50% in the FMT group. The Crohn’s Disease Endoscopic Index of Severity decreased 6 weeks after FMT (*p* = 0.03), but not after sham transplantation (*p* = 0.8). On the contrary, the CRP level enlarged 6 weeks after sham transplantation (*p* = 0.008) but not after FMT (*p* = 0.5) [39]. In Crohn’s disease patients, a temperature rise is often noted as a side effect several hours after FMT. The large-scale study estimates the risk factors of adverse events (AE) in the long term and the potency of FMT in the short term for patients with CD. One month after FMT, 13.6% of mild AEs occurred, including more frequent episodes of defecation, fever, abdominal pain, flatulence, hematochezia, vomiturition, bloating, and herpes zoster. No AEs were observed beyond 1 month. Thus, 1 month could be suggested to establish short-term and long-term AEs of FMT [40]. Other adverse events contain gastroenteritis, upper gastrointestinal tract bleeding, paresthesia, transient sore throat and head-ache, hypotension, herpes zoster, E. coli bacteremia, flares of UC or enteritis, colon micro-perforation, norovirus transmission and significant weight gain, peritonitis, pneumonia, cytomegalovirus infection without donor screening, and so on [41]. Inhalation pneumonia following vomiting occurred after treatment with FMT via nasojejunal tube [34]. Thus, the effectiveness of FMT in CD is still under consideration and requires further study. FMT is a safe and efficient treatment for CD. Besides promising beneficial impact, the next opening issues such as FMT-related donor screening, and feces preparation and use, are still not standardized, and the exact microbial recovery mechanism and long-term safety likelihood remain unrevealed. The future trends of FMT may include sealed frozen FMT agents, which can be orally applied, avoiding invasive procedures and reducing the costs and associated risks [41].

## 5. Irritable Bowel Syndrome

In irritable bowel syndrome (IBS), patients exhibit a reduced variety of gut microbiota, a magnification in the number of *Enterobacteria*, and a relatively low level of *Bifidobacteria* and *Lactobacilli* [42]. Insufficient butyrate production and excess acetate and propionate production in IBS patients are commonly associated with bloating and abdominal pain. There are several prospective and well-controlled studies on the effectiveness of FMT in IBS treatment. In Norway, 90 patients who had IBS with diarrhea (IBS-D) or mixed IBS (IBS-M) were chosen to receive either FMT or a placebo in a single-blind RCT. After 3 months, a clinical improvement (stated as >75-point reduction on the IBS severity scoring system (IBS-SSS)) was noted in 43% of the patients in the placebo group and 65% of the patients in the FMT group (mixed material from two donors), which constitutes a significant difference [43]. In contrast, Halkjær et al. published a larger significant decrease in IBS-SSS (−125.71 ± 90.85) in the placebo group after 3 months, compared with the FMT group that received FMT-capsules for 12 days [44]. However, patients receiving FMT in capsules exhibited a higher microbial diversity, but the symptomatic improvement was higher in the patients of the placebo group [44]. Holvoet et al. examined 64 patients with IBS without constipation [45]. The patients were placed in single-blind randomized trials at a ratio of 2:1 to receive FMT via colonoscopy from two donors (experimental group) or FMT from their feces (control group). There was a significant decrease in discomfort, abdominal pain, and bloating in the experimental group, as compared to the control group. Microbiome analysis revealed that patients receiving effective FMT had a higher baseline concentration of Streptococcus, as well as a higher enrichment of the entire gut microbiome than non-responders [45]. A double-blind, placebo-controlled RCT on the effectiveness of FMT in capsules in patients with IBS-D was carried out by Aroniadis JC et al. in three centers in the USA [46]. Patients were randomly assigned (1:1) in blocks of 4 with a computer-generated randomization sequence to apply 75 capsules with either donor stool (each containing 0.38 g) or 75 capsules of a placebo for 3 consecutive days (25 capsules per day). After 12 weeks, the effectiveness of treatment did not differ significantly in either group, as assessed by the IBS severity index. On this basis, the authors concluded that the effectiveness of FMT in patients with IBS requires further study [46]. 

Recently, the results of the aforementioned studies were pooled in a meta-analysis. A recent meta-analysis of four [47] and five RCT [48] showed that FMT did not improve global IBS symptoms compared with the placebo. On the other hand, recent meta-analyses assessed separately data from single-arm trials (SATs) and RCTs. In SATs, 59.5% (95% CI 49.1–69.3) of IBS patients showed remarkable enhancement. In RCTs, there were no changes between FMT and control in improvement (RR = 0.93 (95% CI 0.50–1.75) or differences in the IBS-SSS [49]. However, the mode of delivery may be a key issue; the classic FMT shows promise, while capsule delivery does not. The benefits of single-dose FMT using colonoscopy and nasojejunal tubes in comparison with autologous FMT for placebo therapy and a decrease in the likelihood of improvement of the multiple-dose capsule were observed in sub-analysis [47,48].

Benno et al. considered FMT in the form of anaerobic cultivated human intestinal microbiota (ACHIM) via the upper gastrointestinal route using endoscopy to achieve a treatment response in 50 IBS patients. A decrease in the number and severity of symptoms, regardless of changes in basic SCFA composition, was observed in most cases: 32 patients had a 50-point reduction of IBS-SSS and 21 had a 100-point IBS-SSS [50]. El-Salhy et al. reported the first placebo-controlled RCT in IBS to use gastroscopy to reach the distal duodenum and to compare the efficacy of two different doses of donor stool in a fecal solution of a remarkably high density (30 g FMT and 60 g FMT in 40 mL saline) [51]. The investigation included more patients (165 patients with IBS) and resulted in higher FMT response rates in comparison with previous studies [52]. Response, defined as a diminution of 50 points or more in the IBS-SSS after 3 months, corresponding to a symptom improvement of 10%, was achieved by 77% of patients in the 30 g FMT group, 89% of patients in the 60 g FMT group, and 24% of patients in the placebo group (who received 30 g of their own stool). Symptom remission, defined as an IBS-SSS score improvement of at least 175 points, was achieved in 35% of patients receiving 30 g of stool, 47% of patients receiving 60 g of stool, and 6% of patients receiving placebo [51].

Thus, despite evidence of the effectiveness of FMT in IBS treatment, it is recommended that this problem is studied further, and the profile of the intestinal microbiome before and after the procedure is examined. Researchers should also consider which gut microbiota changes are associated with clinical improvement.

## 6. Microscopic Colitis

Microscopic colitis (MC) is an inflammation of the large intestine, diagnosed in the presence of microscopic alterations of colonic mucosa that can cause watery diarrhea and cramping in patients. Lymphocytic colitis (LC) and collagenous colitis (CC) are two types of MC; it is hard to identify whether these are the different expressions of one unique disease or if they are distinct conditions [53]. A small portion of patients (1–6%) with MC are resistant to medical treatment. The notion that FMT may be effective in MC was put forward. A recent study assessed the impact of repeated FMT in a patient resistant to medical treatment CC who presented with some symptoms, for example, profuse diarrhea and deep weight loss. It was found that the patient remained in remission for 11 months after 3 courses of FMT [54]. In contrast, another study reported the development of new-onset MC in a UC patient after FMT [55]. The influence of three repeated FMTs (day 0, 14 days, 28 days) in CC patients (*n* = 9), using two stool donors was investigated. The primary endpoint (remission at 6 weeks, defined as <3 stools whereof <1 watery stool per day) was achieved by 2 of these patients, and by 1 at 8 weeks. FMT resulted in a raised number of lamina propria lymphocytes, possibly indicating an initial mucosal immune response [56]. Further studies would be necessary to characterize this subset and to understand if these results can be generalized to all patients with microscopic colitis.

## 7. Functional Constipation

Functional constipation is also associated with intestinal dysbiosis, although there is significantly less evidence for gut microbiota abnormalities in such cases compared with other diseases. An RCT with the inclusion of 60 patients studied the effectiveness of FMT for constipation with slow intestinal transit [57]. Patients who received FMT by introducing donor material through a nasogastric tube showed a more significant improvement in symptoms and stool consistency, as well as an accelerated intestinal transit when compared with patients from the control group. Another RCT reported a clinical remission rate of 15%, and an efficiency rate of 12% after 3 courses of FMT through gastroscopy in patients with chronic functional constipation [58]. Furthermore, FMT treatment increased gastrointestinal motility and peristalsis, and increased serum NO and 5-HT levels, which were accompanied by higher abundances of *Prevotella* and *Acidaminococcus* [58]. These data find confirmation in two prospective studies were a reduction in clinical cure rate over time and higher efficacy of FMT in combination with the prebiotic in patients with chronic constipation were observed [59,60]. Besides promising results, the FMT treatments were quite intense and invasive, with patients receiving up to 18 nasoduodenal FMTs over 3 months [60]. These limitations of dosing frequency and nasal tube placement could be addressed by using FMT capsules. Furthermore, the potency of the fecal microbiota in the FMT can be debated as the glycerol, used to protect the microbiota from freezing, which could have a laxative effect [61]

## 8. Antibiotic-Associated Diarrhea (AAD)

The use of many antibiotics can be accompanied by the development of intestinal dysbiosis, which is often clinically manifested in diarrhea. One study compared the effectiveness of multicomponent probiotics and FMT in patients with AAD [62]. It was concluded that FMT promotes rapid IM recovery and improvement in symptoms, whereas treatment with a multi-component probiotic slowed IM recovery. 

## 9. Immune Checkpoint Inhibitor-Associated Colitis and Gastrointestinal Cancers

Immunotherapy has changed the area of oncology, improving long-term survival in patients across various cancer types. Therapies with immune checkpoint inhibitors (ICI) targeting cytotoxic T-lymphocyte-associated antigen 4 (CTLA-4), programmed cell death protein 1 (PD-1), and programmed cell death ligand 1 (PD-L1) are associated with increased T cell activation and effective anti-tumor immune responses [63]. They have been coupled with several adverse events, including immune checkpoint inhibitor-induced colitis. A recent case series reported the preliminary data of immune checkpoint inhibitor-associated colitis successfully treated with FMT, with the reconstitution of the gut microbiome diversity and the induced complete resolution of clinical symptoms [64]. Following FMT, there was a substantial decrease in CD8+ T-cell density with a concomitant increase in mucosal CD4+ FoxP3+ in one of the patients, offering a potential mechanism through which FMT could abrogate immunotherapy-associated toxicity [64,65]. Moreover, after the establishment of the impact of gut microbiota in determining the reply to immune checkpoint inhibitors, the interest in FMT as a therapeutic option in the management of cancer has risen [66]. To date, only a few studies—most of them with preclinical design—have investigated FMT in gastrointestinal cancer, including colorectal cancer, hepatocellular carcinoma, and pancreatic cancer [67,68,69].

## 10. Chronic Liver Disease

At the present stage, chronic diffuse liver diseases, such as chronic viral hepatitis (CVH), non-alcoholic (NAFLD) and alcoholic liver disease (ALD), are one of the leading causes of morbidity and mortality [70,71]. Despite the breakthrough in recent decades in the management of most forms of CVH, the effectiveness and variability of treatment for other causes of the chronic liver disease remain insufficient. Given that gut microbiota plays a previously invaluable role in the progression and development of most forms of chronic liver disease, it is reasonable to assume that the use of FMT may be an important therapeutic approach in integrated treatment [72].

ALD is part of the clinical and histological complex, which includes fatty liver disease, alcoholic hepatitis (AH), alcoholic cirrhosis, and its complications. AH has a rapid onset and in severe cases can lead to acute/chronic liver failure, when the risk of short-term mortality is 20–50% [73], and 1-year mortality or the need for a liver transplant for nearly 60% [74]. Glucocorticosteroids, abstinence from alcohol, and liver transplantation for patients who do not respond to drug therapy are the main treatment options with proven long-term benefits. Recently, FMT has been studied as a potential treatment for ALD. Philips et al. studied the effectiveness of a 7-day course of FMT in patients who did not receive steroids. Significant improvement was found for the 1-year survival rate in the FMT group (87.5%), compared with the control group (33.3%) [75]. In fact, post-FMT improvement for the relative abundance of *Proteobacteria* and *Actinobacteria*, as well as the species-level reduction of pathogenic *Klebsiella pneumoniae* and an increase in beneficial species (*Enterococcus villorum*, *Bifidobacterium longum*, and *Megasphaera elsdenii*) were observed [75]. A larger retrospective study of 51 patients found that FMT was associated with decreased rates of hepatic encephalopathy (HE), and higher rates of survival at 3 months. In general, the survival rate in FMT groups was assessed at 75%, which was two times higher than in the corticosteroid, nutrition, and pentoxifylline groups where this parameter was 38%, 29%, and 30%, respectively [76]. However, although the results of the study are encouraging, they should be interpreted with caution due to the retrospective design and the unclear assignments of each treatment group [72]. The preliminary results from the first RCT (NCT 03091010) involving the comparison patients (*n* = 112) with severe active AH, treated with glucocorticosteroids or FMT, showed promising results, with an improved 90-day survival rate in the group receiving FMT [73]. Given the poor prognosis of severe AH, and the limited number of treatment alternatives available, further high-quality studies on FMT with greater applicability are certainly warranted to address many unsolved issues.

Primary sclerosing cholangitis (PSC) is a chronic, cholestatic liver disease of mixed etiology, leading to damage to the intrahepatic and extrahepatic bile ducts. Within 10–15 years of diagnosis, more than 50% of patients require a liver transplant [77]. Approximately 70 to 80% of PSC patients also have a co-existing diagnosis of IBD, usually UC [78]. Several theories consider the gut microbiota changes closely associated with the pathogenesis of PSC [79]. One theory about the pathogenesis of the condition is that bacteria from the inflamed intestinal mucosa translocate via the portal venous system into the liver, and stimulate an adverse immune response within the biliary system [80]. Previous studies have found the most pronounced association with *Veillonella, Streptococcus* and *Enterococcus*, compared with other species [81]. Unfortunately, nowadays, no effective treatment has been suggested. However, stemming from the microbiome role, several antibiotics—specifically vancomycin [82] and FMT—have shown promising results [83]. In 2018, the first case report described the usefulness of weekly FMT from a healthy donor for the treatment of recurrent acute bacterial cholangitis in a PCS. After FMT, the patient’s total bilirubin and alkaline phosphatase (ALP) decreased, and remission lasted for over a year [84]. A recent pilot study reported the outcomes of the use of FMT in ten patients with PSC, and an increase in ALP of more than 1.5× the upper limit of normal and concurrent IBD. Since this was the first study in patients with PCS, the safety concerns were the primary endpoint. Overall, 30% (3/10) of the participants experienced a reduction in ALP by at least 50%, with the relative abundance of those bacteria present in the stool microbiota negatively correlating with ALP levels post-FMT, but not pre-FMT [85]. Future larger studies are needed to confirm the efficacy of FMT in PSC.

Hepatitis B is a viral infectious disease that affects the liver and proceeds in an acute or chronic form. The WHO estimates there were 296 million people globally living with chronic hepatitis B in 2019 [86]. New generation technologies have identified the quantitative and qualitative disturbances in the intestinal microbiota and provided the pathognomic basis for the future use of FMT for HBV-related diseases [87]. In a small prospective study, monthly FMT treatment showed a reduction in hepatitis B e-antigen (HBeAg) titers in two of five patients with persistently positive HBeAg, despite more than 3 years of antiviral therapy. However, this effect was not observed in any of the 13 patients in the control group [88]. Another small study confirmed the findings of this previous one. In 16.7% of HBeAg-positive patients on antiviral treatment (AVT) for more than 1 year, antigen clearance was observed after an additional six cycles of FMT via gastroscope at four weekly intervals. In a single AVT, a lack of effect was found (*p* = 0.188) [89]. Preliminary data are quite optimistic, as this may reduce the need for lifelong antiviral therapy and, along with reports of good tolerability of FMT in patients and a small number of mild side effects, this may open a new niche in the treatment of viral hepatitis [90].

Non-alcoholic fatty liver disease NAFLD is a clinicopathological syndrome that includes hepatic steatosis (accumulation of triacylglycerols in the liver over 5% of its weight), steatohepatitis (NASH), and fibrosis which may progress to cirrhosis [91,92,93]. Preclinical studies in obese mice found that FMT reduced weight gain and NAFLD activity score, as well as intrahepatic lipid accumulation and intrahepatic pro-inflammatory cytokines 8 weeks post-FMT [94]. Similarly, mice transplanted with feces from human NASH patients showed an increased hepatic steatosis and inflammatory cell infiltration compared with those transplanted with feces from healthy human controls [95]. To date, FMT trials in human subjects have been limited to obese adults with metabolic syndrome (without defined NAFLD), but at least two RCTs examining FMT in adults with biopsy-confirmed NASH are actively recruiting subjects [96]. Currently, only one pilot RCT for patients with NAFLD assesses the effects of allogenic (*n* = 15) or an autologous (*n* = 6) FMT delivered to the distal duodenum. FMT did not improve IR, as measured by HOMA-IR or the hepatic proton density fat fraction, but it did have the potential to reduce small intestinal permeability in patients with NAFLD [97]. The most important limitation of this study is the different methods that were used for NAFLD confirmation (biopsy, FibroScan, and MR elastography). In a small proof-of-concept RCT, allogenic FMT using lean vegan donors, compared to autologous donors, in individuals with fatty liver showed an effect on gut microbiota, which is associated with beneficial changes in plasma metabolites, and the improvement of histological (necro-inflammation score) and liver gene expression (*ARHGAP18*, *RECQL5*) in liver biopsies [98]. However, the study was prematurely terminated due to a slow recruitment rate. 

NASH is a major cause of liver cirrhosis and hepatocellular carcinoma (HCC); both primary indications for liver transplantation. End-stage liver cirrhosis can lead to recurrent hepatic encephalopathy (HE), which is often associated with the development of intestinal dysbiosis. The possible beneficial effect of FMT for HE has been evidenced in animal studies. FMT enables protective effects in HE rats with CCl4-induced acute hepatic failure, improves cognitive function, and reduces the liver function indexes. Moreover, FMT prevented hepatic necrosis and intestinal mucosal barrier damage, leading to the hepatic clearance of serum ammonia levels, a reduction of intestinal permeability, and the downregulation of TLR4 and TLR9 expression [99]. In a small open-label study, 20 male outpatients with cirrhosis and hepatic encephalopathy were examined and randomly divided into 2 groups. In the first group (experimental group), patients received broad-spectrum antibiotics for 5 days, and then FMT (by enema) from a single donor. Standard therapy was carried out in the second group (control group) [100]. Hepatic encephalopathy recurred in 5 of 10 patients who received standard care, but in none of the 10 patients who received FMT. In addition, the patients in the experimental group exhibited improvements in cognitive function, as well as a relative increase in Lactobacillaceae and Bifidobacteriaceae. A recent study determined the long-term impact of FMT versus standard of care (SOC) over more than 1 year on cognition, hospitalizations, and HE, by extending the results of previous RCTs [100,101]. SOC therapies for the condition include bowel purgatives (in particular, lactulose) and nonabsorbable antibiotics (i.e., rifaximin). FMT was well tolerated and had a long-term safety profile, without infections or the need for new antibiotic therapy. During a long-term follow-up, there were significantly more hospitalizations and episodes of HE in the SOC group compared with the FMT group. In the SOC arm, there was a total of 10 hospitalization (2 participants: 2 events; 6 participants: 1 event) and 8 HE events (2 participants: 2 events; 4 participants: 1 event) compared with 1 hospitalization (*p* = 0.05) and an absence of HE episodes (*p* = 0.05) in the FMT arm respectively [101]. Thus, many questions about the efficacy and safety of FMT in HE remain unanswered, for example: the duration of treatment and the number of procedures required, and the problem of selecting a universal donor [72]. Several RCTs are actively recruiting (NCT02862249, NCT03796598, NCT03439982) patients with HE and cirrhosis, which could help to address unanswered clinical questions in the near future.

## 11. Acute Pancreatitis

Presently, only one case for the efficacy of FMT in moderately severe acute pancreatitis has been reported, which was complicated with a severe *Clostridium difficile* infection. The patient was a 51-year-old man from China who suffered from diarrhea in his course of acute pancreatitis. After obtaining informed consent, the patient’s treatment was started with FMT, instead of metronidazole as suggested for routine practice. The dynamics revealed the disappearance of diarrhea in 5 days post-FMT, and complete recovery according to colonoscopy in 40 days after discharge. Based on this finding, a group of scientists suggested that FMT may be a new treatment option for *Clostridium difficile* infections in patients with acute pancreatitis [102]. Currently, the impact of FMT in patients with pancreatitis is under active investigation and three RCTs have been registered (NCT03015467, NCT02318134, and NCT02318147).

## 12. Non-Gastroenterological Diseases: COVID-19

To the best of our knowledge, there have been no completed RCTs on the safety or efficacy of FMT in patients with COVID-19. However, the first evidence that FMT may be beneficial for patients with COVID-19 was reported in two clinical cases by doctors from Imperial College London and the Medical University of Warsaw [103]. The first case involved an 80-year-old subject who had pneumonia and sepsis (blood poisoning) on top of recurrent *Clostridium difficile* infections: he was given remdesivir and plasma-containing antibodies to SARS-CoV-2 (‘convalescent plasma’). Unexpectedly, his COVID-19 symptoms cleared up two days after the transplant without further worsening of his pneumonia [103]. The second case involved a 19-year-old individual with UC who was being treated with immunosuppressant drugs. He was admitted to the hospital because of recurrent *Clostridium difficile* infections. He was treated with antibiotics and given a stool transplant to prevent further recurrence. Fifteen hours later, he developed a suspected COVID-19 infection, which was confirmed by a positive swab test. Subsequently, other than two isolated episodes of fever, his COVID-19 symptoms cleared up. This second patient was not given any other medication to specifically treat his COVID-19 [103]. At present, Zhang et al. are investigating the effectiveness of washed microbiota transplantation in patients with COVID-19 for improving mortality rates, and quality of life of patients with COVID-19 [104]. This study has a blinded and placebo-controlled design. Patients enrolled in the study in addition to standard therapy received either a washed suspension of microbiota or placebo through a nasogastric/nasojejunal tube, or orally [104].

### 12.1. Psoriasis

Psoriasis is a widespread inflammatory skin disease that is pathophysiologically similar to IBD. It has been found that patients with psoriasis exhibit abnormalities in gut microbiota, in particular, a reduction in the relative abundance of *Akkermansia mucinophila* and a three-fold increase in the *Bacteroidetes*/*Firmicutes* ratio [105]. Effective psoriasis treatment is usually associated with a marked improvement in the composition of gut microbiota. The first clinical evidence for FMT efficacy in psoriasis was described in a 36-year-old Chinese male, who had suffered from psoriasis for 10 years and IBS for 15 years. FMT was initially performed by upper endoscopy, and repeated after 5 weeks via colonoscopy. Post-FMT, there was a reduction of serum TNF-α and intestinal symptoms, as well as an improvement of the Psoriasis Area and Severity Index (PASI), the Dermatology Life Quality Index (DLQI), and histological examination as compared to the baseline condition were observed [106]. A 6-month, double-blind, placebo-controlled RCT of the effectiveness and safety of FMT in patients with psoriatic arthritis is currently underway [107].

### 12.2. Multiple Sclerosis 

Multiple sclerosis (MS) is a chronic autoimmune demyelinating disease that causes severe neurological changes, for which there is an extant lack of highly effective treatment options. Many patients with multiple sclerosis have gastrointestinal symptoms and gut microbiota changes in comparison with healthy people [108]. In one experiment, the inflammatory response in mice with autoimmune encephalomyelitis (AE) decreased when bacterial strains producing butyrate were administered [109]. On the other hand, feces transplantation from patients with MS could precipitate an MS-like autoimmune disease in mice [110]. Li et al. tested FMT in mice with experimental AE, a mouse model of MS. FMT can rectify altered gut microbiota and led to a reduced activation of microglia and astrocytes, and conferred protection on the blood–brain barrier (BBB), myelin, and axons in experimental AEs [111]. The first case report in three people with MS described a short-term improvement of neurological symptoms after FMT for constipation [112]. A recent study suggested that FMT administered for over 10 years has a potential long-term benefit on MS disease progression [113]. This proof-of-concept study suggests that FMT might be an emerging treatment in relapsing-remitting MS. FMT interventions were associated with increased abundances of putative beneficial stool bacteria and short-chain-fatty-acid metabolites, which were associated with increased/improved serum brain-derived-neurotrophic-factor levels and gait/walking metrics [114]. The main limitation was the participation of only one patient, but the findings may constitute an important background for scientific rationale, and help design future RCTs assessing FMT in MS patients. Therefore, there is an extant view that MS can be treated by FMT, just as UC can. Three studies on the effectiveness of FMT in patients with multiple sclerosis are currently ongoing.

### 12.3. Parkinson’s Disease (PD)

PD is an intractable neurodegenerative disease that is often associated with gastrointestinal disorders such as constipation, IBD, and IBS. PD patients also appear to have increased intestinal permeability [115] and small intestine bacterial overgrowth [116]. The gut microbiome of patients with PD is characterized by an overabundance of *Bacteroidetes*, *Faecalibacterium prausnitzii*, *Enterococci*, *Prevotella*, and *Clostridium*, particularly in severe cases [117,118]. Overall, more pro-inflammatory gut bacteria, such as LPS-producing *Proteobacteria*, and less anti-inflammatory butyrate-producing gut bacteria are found in PD patients [117,119]. 

In one experiment, the transplantation of microbiota from patients with PD in a mouse model led to a worsening of neurological manifestations, whereas gut microbiota depletion in the same model reduced neurological symptoms [120]. Another study showed that a PD mouse model had improved motor function, increased striatal neurotransmitters, and decreased neuroinflammation after receiving feces from healthy mice [119,120]. Furthermore, a healthy mouse donor FMT had neuroprotective effects in PD mice through the suppression of neuroinflammation and a reduction in TLR4/TNF-α signaling [121]. Zhou et al. demonstrated that intestinal microbiota may have a neuroprotective effect. FMT from normal mice with a fasting-mimicking diet to animals with PD has been shown to increase dopamine levels in substantia nigra [122]. 

The first case report assessing the effects of FMT in PD was described in a 71-year-old male patient who presented with 7 years of resting tremor. The patient successfully defecated within 5 min, and maintained daily unobstructed defecation until the end of the follow-up. The patient’s tremor in the legs almost disappeared 1 week after FMT, but recurred in the right lower extremity 2 months after FMT [123]. Xue et al. reported data from the first pilot study for the efficacy and safety of FMT on 15 PD patients. FMT with a preference for colonic rather than the nasointestinal way can relieve the main PD symptoms. Moreover, 2/10 patients from the colonic FMT group in 2-year follow-up achieved satisfactory results. In the nasointestinal FMT group, all beneficial effects terminated after 3 months. FMT was safe, and only five mild and self-limiting adverse events occurred during the study [124]. Preliminary literature suggests that FMT may be a promising treatment option for PD. In summary, it should be noted that the available evidence is currently insufficient and is based on the scanty number of both experimental and RCTs, which necessitates further studies of FMT to assess the safety and the preferable methods of FMT [119].

### 12.4. Autism Spectrum Disorder

Autism spectrum disorder (ASD) is a condition related to brain development that impacts how a person perceives and socializes with others, causing problems in social interaction and communication [125]. ASD is often associated with constipation, bloating, diarrhea, and changes in the gut microbiome [126,127]. Children with ASD usually have a reduced *Bacteroidetes*/*Firmicutes* ratio [128] and increased levels of the genus *Clostridium* [125]. Changes in the microbiome may interact with tryptophan metabolism and contribute to behavior change, but the evidence is inconsistent [129]. 

FMT from children with ASD to germ-free wild-type mice associated with the development of ASD-like symptoms displayed alternative splicing of ASD-relevant genes in their offspring [130]. Another study observed a reduction in oxidative stress markers, primarily glutathione and vitamin C, in the brains of ASD patients [131]. Probiotic/prebiotic treatments showed ameliorative effects; however, lactobacillus had the strongest [131].

A pilot open-label study investigated the effectiveness of FMT in 18 children (aged between 7 and 16 years old) with ASD after a 2-week course of antibiotic treatment. Parallel enhancement in ASD behavior scores and a decrease in intestinal symptoms (bloating, constipation, diarrhea) were observed, and the improvements persisted for more than 2 months after the FMT had been administered. In addition, there was engraftment of donor stool microbiota with an increase in both overall bacterial α-diversity, as well as an abundance of *Bifidobacteria*, *Prevotella*, and *Desulfovibrio* which persisted for more than 2 months post-FMT [132]. These same benefits appeared to be maintained when participants were followed up for up to 2 years after FMT [133]. However, this study was open-label, and there was no comparator arm of patients receiving placebo/autologous FMT, no controlling for diet or nutritional supplements, and a lack of information on adverse events in the long-term follow-up. An open-label randomized waitlist-controlled trial showed a significant improvement of the Childhood Autism Rating Scale in the FMT group, as compared to the waitlist group after the first procedure (10.8 vs. 0.8%, *p* < 0.001), and shifted the microbiome of ASD patients to a healthy state [134]. Although these observations point to a potential causal link between the microbiome and ASD, the results are preliminary and speculative.

### 12.5. Epilepsy

Epilepsy is a central nervous system (neurological) disorder in which brain activity becomes abnormal, causing seizures or periods of unusual behavior, sensations, and sometimes a loss of awareness [135]. In most cases, the etiology of the disease is unknown, but in some people epilepsy is caused by trauma, stroke, brain tumors, drug and alcohol abuse, or other causes [136]. The composition and distribution of gut microbiota profiles in patients with refractory epilepsy differ from healthy controls. Several studies reported an increased *Firmicutes*/*Bacteroides* ratio and α-diversity, as well as *Ruminococcus*, *Akkermansia*, *Neisseria*, *Coprococcus*, *Methanobrevibacter*, and *Roseburia* [137,138]. Moreover, the abundance of *Bifidobacterium* and *Lactobacillus* was associated with fewer seizures per year [137], and a ketogenic diet reduced the frequency of seizures by modulating the gut microbiota [139].

A recent study found that both chronic stress and microbiome transplanted from stressed to sham-stressed rats accelerated the progression and prolonged the duration of kindled seizures [140]. Olson et al. observed that the transplantation of ketogenic microbiota or the long-term administration of species *Akkermansia muciniphila*, *Parabacteroides merdae*, and *Parabacteroides distasonis* decreased the number of seizures in mice at a higher threshold [141]. He et al. reported a case report in which FMT was used to achieve a remission of intestinal and neurological symptoms in a girl with CD and a 17-year history of generalized epilepsy [142]. During the 20 months of follow-up, three rounds of FMT proved efficacious in preventing the relapse of seizures after withdrawing the sodium valproate [142].

### 12.6. Other Neurological Disorders

Vendrik et al. analyzed studies and case descriptions on FMT in neurological disorders in humans or animal models. From 541 identified studies, 34 were included in the analysis [119]. For stroke, Alzheimer’s disease, and Guillain–Barré syndrome, only studies with animal models were identified. These studies suggested a potential beneficial effect of healthy donor FMT. In contrast, one study with an animal model for stroke showed increased mortality after FMT [119]. Only one study was identified for Guillain–Barré syndrome. It should be noted that it is not known whether the previous positive experimental results will be reflected in the treatment of patients. To date, several RCTs are scheduled, or are in the stage of active recruitment to validate the use of FMT for the treatment of the above-mentioned neurological disorders [124].

### 12.7. Metabolic Syndrome/Obesity

The development of metabolic syndrome is usually associated with changes in the gut microbiota. In recent times, one of the most essential aspects of obesity has been considered to be a modification in bacterial aches in the human gut [143,144]. Metagenomic studies and an analysis of 16S ribosomal DNA revealed significant differences in the composition of gut microbiota, and the number of genes when the feces of obese subjects and people of a healthy weight were compared [145]. Without diminishing the role of heredity and environmental factors, the gut microbiota makes a significant contribution to the development of metabolic disorders and obesity, modulating the cascade of host enzymatic reactions, interacting with receptors directly and/or using its own metabolites and signaling molecules [146].

It is obvious that maintaining homeostasis and normal metabolism is impossible without restoring the diversity of normal associations of gut microbiota. Despite the proven effect of diet, pre- and probiotics, further research is needed in order to develop differentiated regimens for the impact on gut microbiota, and thus achieve an improvement in metabolism and lose weight [147,148]. FMT may be considered as a potential therapeutic strategy for the treatment of obesity in the future. An early pilot study split 18 patients into two groups: patients who received allogeneic FMT from lean donors (*n* = 9) and obese patients who received autologous FMT (*n* = 9). The group receiving allogeneic FMT displayed improved insulin sensitivity after 6 weeks [149]. However, a subsequent larger study (*n* = 38) showed that allogeneic FMT (*n* = 26) failed to reduce insulin resistance, compared with autologous FMT (*n* = 12) after 18 weeks, and correlated to a lack of overall change in the composition of the intestinal microbiota [150]. FMT with oral capsules from lean donors to obese patients was tested in double-blind, placebo-controlled studies by Allegretti et al. It was shown that FMT did not affect the patient’s body mass index (BMI) and area under the curve (AUC) of GLP1, but helped to reduce the concentration of taurocholic acid. Patients who received FMT had sustained shifts in microbiomes associated with obesity toward those of the donor [151]. The same group reported the secondary analysis of a previous RCT, with the analysis of post-prandial glucose and insulin levels. There was a significant change in glucose AUC at week 12, compared with the baseline, and in the insulin AUC at week 6 compared with the baseline in the FMT group versus placebo [152]. Weekly administration of FMT capsules in a double-blind randomized placebo-controlled study for adults with obesity resulted in gut microbiota engraftment in most recipients for at least 12 weeks. Despite the lack of metabolic parameters, changes such as insulin sensitivity, HbA1c, body weight, and body composition by DXA were assessed [153]. Zhang et al. have analyzed and compared data on the use of FMT in systemic review. Studies reported mixed results about improvement in metabolic parameters. Two studies reported improved peripheral insulin sensitivity at 6 weeks in patients receiving donor FMT versus patients receiving the placebo [154]. No differences in fasting plasma glucose, hepatic insulin sensitivity, BMI, or cholesterol markers were observed between the two groups across all included studies [154]. FMT has significantly increased the number of species such as *Roseburia intestinalis*, *Akkermansia muciniphila*, and *Clostridium* species. [154]. The most recent meta-analysis, with the inclusion of 6 RCTs and a total of 154 patients, evaluated the role of FMT from the lean donor(s) compared with any form of placebo (sham, saline, autologous FMT, or placebo capsules) for the treatment of obesity and metabolic syndrome. It was found that 6 weeks post-FMT, the level of HbA1c was significantly reduced. However, no difference was found for anthropometric parameters that characterize obesity at 6–12 weeks after the procedure [155].

It is believed that the responses of patients with metabolic syndrome to modification of the gut microbiome may depend on the microbiota’s initial state and diet. Guirro et al. evaluated the effect of a hypercaloric diet on gut microbiota, and this was combined with antibiotic treatment to deplete the microbiota before FMT to verify its effects on gut microbiota-host homeostasis in rats. An HFD affected the gut microbiome and after the antibiotic therapy and subsequent use of FMT, the number of *Bacteroidetes*, *Firmicutes* was increased to the level that was before the antibiotic therapy [156]. The largest recent RCT included 90 participants to evaluate the efficacy and safety of diet-modulated autologous FMT for the treatment of weight regain after the weight-loss phase (DIRECT PLUS trial) [157]. The participants were randomly assigned to groups that received 100 capsules containing their own frozen fecal microbiota or placebo, until month 14. It was found that FMT administrated in the regain phase might preserve weight loss and glycemic control, and was associated with specific microbiome signatures [158]. A high-polyphenols, green plant-based or Mankai diet better optimized the microbiome for an autologous FMT procedure [158].

Preliminary studies showed a promising beneficial effect of FMT, manifested by improved insulin sensitivity, glycemic control, and reduced chronic systemic inflammation [159]. However, high-quality well-powered RCTs with longer follow-up are urgently needed to highlight the benefits of FMT as a viable option for patients with obesity and metabolic syndrome in the future.

### 12.8. Graft-Versus-Host Disease 

Graft-versus-host disease (GvHD) is a syndrome characterized by inflammation in different organs. GvHD is commonly associated with bone marrow transplants and stem cell transplants. Allogeneic hematopoietic cell transplantation (alloHCT) is a potentially curative strategy for patients with selected blood diseases. Complications from the procedure comprise most of all infections and GvHD, which are the major causes of morbidity and mortality (45% of attributable deaths) apart from relapse [160]. The first-line therapy in acute GvHD is systemic administration of high-dose glucocorticoids, but only 40–60% of patients respond to this treatment depending on the grade of severity of the disease [161]. At present, there is no established standard-of-care second-line therapy. The high mortality rate of steroid-refractory/dependent (sr/d) acute GvHD, especially in patients with grade III-IV lower gastrointestinal tract involvement, is a major drive for exploring novel therapeutic strategies [161,162]. As the patients with gut GvHD are often colonized with antibiotic-resistant bacteria (ARB), there are pioneer studies of experience performing FMT in patients with acute or chronic GvHD, co-colonized with ARB. These studies have shown a good efficacy of FMT in the treatment of GvHD and decolonization of the GI tract from ARB [160,161]. 

We suggest that the treatment with FTM may improve the chances for successful convalescence of these patients. Still, there are very few studies evidencing the results of such a treatment method. Future research should focus on randomized controlled trials for the implementation of this therapy in clinical practice, and understanding the side effects.

## 13. Conclusions

Despite advances in gut microbiota research, there have been few controlled studies investigating the effectiveness of therapeutic interventions that can truly modify the microbial population in the human intestine. A similar gap in knowledge affects FMT. In modern medicine, FMT was first effectively used to treat patients with recurrent *Clostridium difficile*-associated colitis, which is currently the only officially approved ground for its use. Regulations and classifications of FMT, however, have evolved over time. Active participation in the field of contribution for FMT research vary between different countries and regions (Figure 1 and Figure 2). 

The Medicines and Healthcare Products Regulatory Agency (MHRA) in the UK, and the FDA in the USA, currently regulate FMT as a medicinal product. Within mainland Europe, in 2014, the European Commission advised that FMT is a ‘combined substance’ consisting of both human and nonhuman components. On the basis that the active components of FMT are likely to be associated with nonhuman components, it was considered to fall outside of the European Tissues and Cells Directive, and therefore should be regulated at the local/national level [66].

Nevertheless, the effectiveness of FMT is currently being studied intensively, and refined for the treatment of several digestive and non-digestive diseases (Table 1).

## Figures and Tables

**Table 1:** Diseases for which the effectiveness of FMT is being investigated.

Disease	Study Type	Key Findings
Ulcerative colitis (UC)	RCT	Steroid-free remission, an endoscopic and clinical response in 20–32% patients after FMT.
Pouchitis	Series of Cases	Betterment in gut motility and a trend for improvement in abdominal pain post-FMT. Clinical remission rate followed FMT 21.5–32%. Poor endoscopic response.
Crohn’s Disease (CD)	Small open-label studies	Overall clinical remission of 37% and an overall clinical response of 54%
Irritable bowel syndrome (IBS)	RCT	In single-arm trials 59.5–67% (95% CI 49.1–69.3) of IBS patients showed remarkable clinical improvement which stated as >75 points reduction on IBS severity scoring system (IBS-SSS). In RCTs, there were no changes between FMT and control in improvement.
Microscopic colitis (MC)	Case reports	Poor data, further studies needed.
Functional Constipation	RCT	FMT treatment increased gastrointestinal motility and peristalsis. Clinical remission rate up to 20% post-FMT.
Immune checkpoint inhibitors associated colitis	Series of Cases	Preliminary data suggested that FMT lead to reconstitution of the gut microbiome diversity and induce complete resolution of clinical symptoms.
Alcoholic liver disease (ALD)	Small open-label studies Single RCT	FMT was associated with decreased rates of hepatic encephalopathy (HE) and rates of short-term survival higher than 75%.
Primary sclerosing cholangitis (PSC)	Series of Cases Pilot study	The reduction in alkaline phosphatase by at least 50% overall in 30% of participants.
Hepatitis B	Small open-label studies	The reduction in hepatitis B e-antigen (HBeAg) clearance.
Non-alcoholic fatty liver disease (NAFLD)	Preclinical studies	FMT reduced weight gain, NAFLD activity score, as well as in intrahepatic lipid accumulation and intrahepatic pro-inflammatory cytokines.
Non-alcoholic fatty liver disease (NAFLD)	Pilot studies RCT in active recruitment	No improvement of insulin resistance as measured by HOMA-IR or hepatic proton density fat fraction. Beneficial changes in plasma metabolites, improves histological (necro-inflammation score) and liver gene expression (*ARHGAP18*, *RECQL5*).
Acute pancreatitis	Case report	Poor data, further studies needed.
COVID-19	Case report	Poor data, further studies needed.
Psoriasis	Case report RCTs ongoing	Reduction of serum TNF-α and intestinal symptoms, as well as improvement of psoriasis area and severity index (PASI), dermatology life quality index (DLQI) in case report.
Multiple Sclerosis	Case report RCTs ongoing	Short-term improvement of neurological symptoms post FMT in case reports.
Parkinson’s Disease	Case reports RCTs planned	Short-term improvement of neurological symptoms post FMT in case reports.
Autism spectrum disorder	Case reports Uncontrolled Pilot Study	Improvement of the Childhood Autism Rating Scale post FMT.
Epilepsy	Preclinical Case reports	Decreased the number of seizures in mice at a higher threshold. Long-term efficacy of FMT in preventing relapse of seizures after withdrawing the sodium valproate.
Metabolic Syndrome/obesity	Controlled Study RCTs ongoing	Preliminary studies showed a promising beneficial effect of FMT, which is manifested by improved insulin sensitivity, glycemic control, and reduced chronic systemic inflammation. Lack of effect for anthropometric parameters that characterize obesity.
Graft-versus-host disease	Controlled Study	Preliminary studies have shown a good efficacy of FMT in the treatment of GvHD and decolonization of the GI tract from antibiotic-resistant bacteria.

## Data Availability

Not applicable.
